# The extended gentle caesarean section protocol—expanding the scope and adding value for the family: a cross-sectional study

**DOI:** 10.1007/s00404-023-06913-0

**Published:** 2023-01-16

**Authors:** Patricia Christoph, Julia Aebi, Lena Sutter, Kai-Uwe Schmitt, Daniel Surbek, Stephan Oelhafen

**Affiliations:** 1 Department of Obstetrics and Gynecology, Bern University Hospital, University of Bern, 3010 Bern, Switzerland; 2 School of Health Professions, Bern University of Applied Sciences, 3008 Bern, Switzerland; 3 Department of Nursing, Insel Gruppe, Bern University Hospital, 3010 Bern, Switzerland

**Keywords:** Gentle caesarean section, Skin-to-skin contact, Breastfeeding, Birth experience, Mother-to-child bond

## Abstract

**Purpose:**

In Switzerland, about one in three children is born by caesarean section (CS). For many women, this means a restricted birth experience, limited observation of the birth process and a restricted involvement. We evaluated an extended gentle CS protocol, which offered early intraoperative skin-to-skin contact and the possibility of observing the delivery of the baby from the abdomen through a transparent drape.

**Methods:**

This is a cross-sectional study incorporating data from a purposely tailored questionnaire and clinical routine data. The extended gentle CS protocol was compared with the gentle CS, which does not allow the possibility of observing the delivery. Data were collected online and analysed by multivariable regression for quantitative data and content analysis for all text responses to open questions, respectively.

**Results:**

193 women completed the questionnaire. Of these, 154 had a gentle CS and 39 had an extended gentle CS. Multivariable regression did not reveal a statistically significant difference for extended gentle CS with regard to satisfaction with childbirth, mother-to-child bonding, or breastfeeding duration. Nevertheless, early intraoperative skin-to-skin contact was associated with the fulfilment of birth expectations. Furthermore, most women who experienced an extended gentle CS would prefer the same procedure for any potential future CS.

**Conclusions:**

Although our study showed no statistically significant difference in satisfaction from using a transparent drape, most women expressed a preference for this technique. We recommend that the option of an extended gentle CS should be offered to all women for whom CS is indicated.

**Supplementary Information:**

The online version contains supplementary material available at 10.1007/s00404-023-06913-0.

## What does this study add to the clinical work

**Table:** 

An extended gentle caesarean section protocol that added the possibility of observing the delivery from the abdomen through a transparent drape was experienced positively by most women included in this study. Thus, it is recommended to offer this option to all women for whom caesarean section is indicated.	

## Introduction

In Switzerland, about one in three children is born by caesarean section (CS) [1]. The ‘traditional’, standard CS tends to limit the woman’s overall experience and choices during childbirth [2]. Parents are unable to observe the birthing process as there is a sterile drape present between the mother’s head and her abdomen [3]. The feeling of maternal involvement in the birth procedure is limited and most mothers have reported an unsatisfactory sense of achievement during the process that can negatively impact the mother-to-child bond [4–7].

When compared to the ‘traditional’ CS, the *gentle* CS is a delivery process that promotes the involvement of the patient and family during the surgical procedure [5, 8, 9]. It imitates certain aspects of vaginal delivery by allowing the mother, e.g. to have early intraoperative skin-to-skin interaction with the baby. Since vaginal deliveries and early skin-to-skin contact are both positively associated with initiation and duration of breastfeeding and mother-to-child bonding [6, 10–12], it is possible that the *gentle CS* may reduce these postpartum difficulties. The gentle CS has also been reported to reduce maternal anxiety and enhance the mother’s sense of involvement in the birth procedure, which empowers her [5].

University hospitals tend to have an increased rate of high-risk pregnancies and, correspondingly, more CS due to maternal and foetal risks. Therefore, to improve the experience for women having a CS, the current *gentle CS* protocol was developed further to an *extended gentle CS* protocol that added the possibility of observing the delivery from the abdomen through a transparent drape. This technique allows to have an early visual contact with the baby. Using a self-administered questionnaire, the aim of the present study was to evaluate patient satisfaction with this *extended gentle CS* compared to the previous *gentle CS*, which only included early intraoperative skin-to-skin contact whenever possible.

## Methods

### Study design

We conducted a single-site, cross-sectional survey study combining clinical routine data and data from a self-administered, web-based questionnaire that was developed for the purpose of this study.

### Questionnaire

The questionnaire consisted of a section with Salmon’s Item List (SIL) German [13, 16] and a section with items developed by an inter-professional team consisting of maternal–foetal medicine specialists, midwives and psychologists. These newly generated items were inspired by existing questionnaires. They comprised items regarding the quality of care provided by healthcare professionals [14], understanding the reasons for the CS, skin-to-skin contact in the operating room, preference for and duration of breastfeeding, quality of bond with the newborn, various childbirth parameters and birth history, and sociodemographic data. Women who had experienced an extended gentle CS were also asked specific questions about the visual experience during the procedure and the women also assessed how their partners experienced the birth. The questionnaire also included some open questions that allowed women to describe their experience.

We used the Content Validity Index (CVI) to quantify both relevance and validity of all questionnaire items [15]. Nine healthcare professionals and experts (four obstetricians, three midwives, two professors in the healthcare domain) rated all items based on relevance and clarity on a 4-point Likert scale and provided comprehensive written feedback that was used to revise the items. Subsequently, an index was calculated that reflected the number of experts who rated an item as *rather* or as *completely* comprehensible or relevant. One item on household income with a low average rating of both clarity (0.29) and relevance (0.57) was excluded from the final questionnaire and not replaced. After dropping this item, the average rating of item clarity was *M* = 0.98 (range 0.88–1.00), and the average rating of relevance was *M* = 0.95 (range 0.75–1.00) [15]. The final version of the questionnaire was available in German only (an English translation is available as supplementary material).

The 12-item short version of Salmon’s Item List (SIL) German [13, 16] was used to assess satisfaction with the childbirth experience. Although the scale is divided into four subscales (‘Fulfilment’, ‘Good emotional adaptation’, ‘Neg. emotional experience’, ‘Physical discomfort’), it is recommended to interpret the short version only with regard to the overall satisfaction with childbirth. Cronbach’s Alpha, reflecting the scale’s internal consistency, was high in the current study: α = 0.92 (95% confidence interval 0.90–0.94, *n* = 193). Variables reflecting the age of the mother at birth, the type of CS (planned, unplanned or emergency), nationality, civil status, and preterm birth were taken from clinical routine data.

### Study population and data collection

All women who had a CS between January 1, 2019 and June 30, 2020 at the University Women’s Hospital in Bern, Switzerland, and who had a gestational age of at least 32 + 0, were identified from the hospital’s database. Women were approached by a letter including both a vanity URL and a QR code, which were linked to the self-administered questionnaire on the Qualtrics platform. The technique (gentle or extended gentle CS) was not mentioned to prevent selection bias. Based on a supplied three-character alpha-numeric code, survey responses could then be anonymously linked with the extracted data from the hospital’s medical information system.

### CS protocols

The current *gentle* CS protocol at our tertiary care hospital contained elements, such as placement of the infant for early intraoperative skin-to-skin contact with the mother if both are clinically stable after paediatric assessment; encouragement of early breastfeeding; and avoidance of separation of mother and infant unless clinically indicated or desired by the mother.

The *extended gentle* CS was introduced in December 2017. The protocol encompasses the additional possibility of visual contact with the baby by observing the delivery as well the cutting of the umbilical cord, using a standard size transparent drape. This option is offered to all women with a gestational age of at least 32 weeks. Inclusion criteria are the desire of the woman/the couple for the extended protocol, no complications to be expected (placenta detachment, increased bleeding), and it has to be ensured that the woman/the couple understands the protocol. Preliminary discussions with women and clarification of whether women would be suitable for such a birth help to minimise the risk of causing any trauma from “watching the caesarean section”. Exclusion criteria are, e.g. emergency CS in general anaesthesia, patients with previous mental illnesses, known foetal anomalies, trauma (after clarification), and difficulties in verbal communication.

### Data analysis

The primary goal of the data analysis was to estimate the potential impact of the extended gentle CS on: (a) the overall satisfaction with the childbirth experience (as assessed by SIL); (b) whether their expectations regarding the CS were met (1 item); (c) mother-to-child bond (2 items); and (d) the duration of breastfeeding (partial or exclusive, in months). We therefore conducted four multivariable linear regressions on these four dependent variables whilst controlling for all potentially confounding demographic, pregnancy- and birth-related variables. Responses to open-ended questions regarding the extended gentle CS were categorised by one researcher (J.A.) using a simple content analysis, with the goal of providing counts of the most frequent responses.

Statistical analyses were performed using R 3.6.3 [17]. For descriptive statistics, groups were compared using Fisher’s exact test or Welch’s two sample *t* test. Multivariable regressions were conducted using base R and the car package to calculate variance inflation factor [18].

## Results

### Descriptive statistics

Of the 1044 women who were invited to participate in the study, 211 women initiated the web survey and 193 completed the questionnaire. Of these 193 women, 154 (80%) had a gentle CS and 39 (20%) had an extended gentle CS (see Table 1). Women who had an extended gentle CS more often had private or semi-private health insurance, a university degree and had a CS that had been planned. In addition, around two-thirds of all women (67.6%) had given birth between 1 and 2 years ago, and the average age of children shows that women who had an extended gentle CS had a somewhat more recent birth. All of these variables were added as potential confounding factors in the subsequent multivariable regressions.

**Table 1:** Demographics and selected pregnancy- and birth-related variables of women with gentle or extended gentle caesarean section (*n* = 193)

	Gentle CS (*n* = 154) *n* (%)	Extended gentle CS (*n* = 39) *n* (%)	p*
Age (years)^a^	33.7 (4.8)	34.7 (3.8)	0.2
Nationality			0.5
Swiss	123 (84%)	30 (79%)	
Other	24 (16%)	8 (21%)	
Native language			> 0.9
German	138 (94%)	36 (95%)	
Other	9 (6.1%)	2 (5.3%)	
Civil status			0.2
Single^b^	49 (33%)	8 (21%)	
Married or registered partnership	98 (67%)	30 (79%)	
Insurance classes			0.037
Private	4 (2.7%)	2 (5.3%)	
Semi-private	14 (9.5%)	9 (24%)	
General	129 (88%)	27 (71%)	
Education			0.004
Compulsory school	3 (1.9%)	0 (0%)	
Vocational training	32 (21%)	4 (10%)	
High school	4 (2.6%)	2 (5.1%)	
Higher vocational training	50 (32%)	5 (13%)	
University of Applied Sciences or equivalent	27 (18%)	7 (18%)	
University degree	31 (20%)	20 (51%)	
Other	7 (4.5%)	1 (2.6%)	
Primiparity	96 (62%)	19 (49%)	0.14
Multiple birth	14 (9.1%)	5 (13%)	0.5
Preterm birth	40 (27%)	5 (13%)	0.090
Type of caesarean section			< 0.001
Planned	57 (37%)	30 (77%)	
Unplanned	58 (38%)	8 (21%)	
Emergency	39 (25%)	1 (2.6%)	
Age child (months)^a^	15.2 (4.9)	13.2 (4.9)	0.031

*p values reflect the test for differences between the two groups, conducting Fisher's exact test (in case of frequencies) or Welch’s two sample *t* test (in case of mean values)

^a^Mean (SD)

^b^Single, widowed, divorced or ‘other’

### Multivariable regressions

Table 2 presents results from four models representing multivariable regressions on satisfaction with childbirth experience (SIL), fulfilment of expectations regarding CS, mother-to-child bond, and breastfeeding duration. Having a planned CS led to more positive birth experiences in terms of both satisfaction with childbirth and fulfilment of expectations. Quality of care—as subjectively assessed by the women on the basis of openness to wishes and needs, quality of information and active involvement—was strongly associated with satisfaction and fulfilled expectations. Moreover, having the option of skin-to-skin contact in the operating room led to women's expectations regarding CS being fulfilled more often.

**Table 2:**
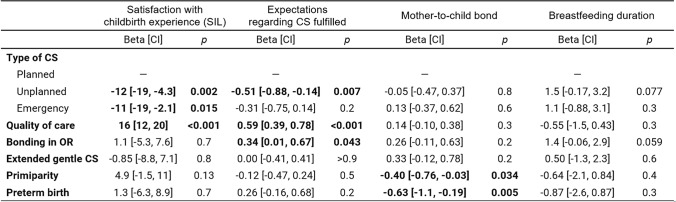
Results from multivariable regression analysis on the association between birth- and pregnancy-related characteristics and satisfaction with childbirth experience (SIL), expectations regarding CS fulfilled, mother-to-child bond and breastfeeding duration (*n* = 193)

*CS* caesarean section, *OR* operating room, *CI* 95% confidence interval

bold values indicate statistically significant effects

The main negative influences on the subjective mother-to-child bond were primiparity and preterm birth. No other variables had a statistically significant impact on breastfeeding duration, although bonding in the operating room trended to have a positive influence. Overall, the extended gentle CS protocol did not show a statistically significant difference with regard to any of these four outcomes.

### Specific questions regarding extended gentle CS

Descriptive statistics of specific questions asked regarding the extended gentle CS and regarding future CS revealed an overall positive picture (Table 3). All women who experienced an extended gentle CS stated that they either would have preferred to see more of the birth (51%) or that the provided exposure felt just right (49%), and none seemed to be overwhelmed by the visual contact. According to these women, only two partners (5.1%) felt they would have preferred to see less of the birth. More importantly, 37 (95%) women who had an extended gentle CS and 81 (53%) women who had a gentle CS would prefer this procedure if they were to have another CS in the future.

**Table 3:** Results from specific questions regarding the extended gentle CS protocol and preference for possible future CS (*n* = 193)

	Gentle CS (*n* = 154) *n* (%)	Extended gentle CS (*n* = 39) *n* (%)
Assessment of the view		
I would have preferred not to see that much of the birth	–	0 (0%)
It was just right how much I saw of the birth	–	19 (49%)
I would have preferred to see more of the birth	–	20 (51%)
Assessment view by the birth partner*	–	
He/she would have preferred to see less of the birth	–	2 (5.1%)
It was just right how much he/she saw of the birth	–	27 (69%)
He/she would have preferred to see more of the birth	–	9 (23%)
I had no birth companion during the caesarean section	–	1 (2.6%)
Preference for possible next caesarean section		
Extended gentle caesarean section	81 (53%)	37 (95%)
Gentle caesarean section	73 (47%)	2 (5.1%)

*According to the surveyed women

In open questions regarding the extended gentle CS, many women described their overall experience as positive (*n* = 18). Women described their experience as: “This gives you at least an impression of a ‘normal’ birth”, “I thought it was great to be able to witness/see the moment of birth like that”, “It's nice to see the baby being taken out of the womb. Would do it again and again!” or “Being somewhat passive, it's nice to be able to see something through the window [in the drape] after all”. Other women highlighted the first visual contact with the baby (*n* = 7), in particular when immediate physical contact was not possible. Importantly, a quarter of the women (*n* = 9) reported that they had seen too little of the birth due to the angle or because it had happened so fast. As a result, they did not see the child until it was expelled or they barely remembered the brief moment. Accordingly, the most frequent suggestions for improvement were that the woman’s head is raised (*n* = 4) and that she should be able to look through the transparent drape for longer (*n* = 6).

Of all women who would like to have an extended gentle CS (in case of a possible next CS) (*n* = 113) mentioned the overall birth experience (*n* = 45) and the visual contact (*n* = 36) as reasons. Many women stressed that they would have wanted to see their children immediately, “It was difficult to hear the child, but not to see anything”. Many women also said they hoped they would be "closer" to what was happening, making the birth more “real”. Other women associated an extended gentle CS with the feeling of being more involved (*n* = 43). They wished to have “More control and participation”. “You feel the pressure, the manipulation and don't know what exactly is going on. I would have liked to know what happens”.

Women who had a gentle CS *without* visual contact and who said they would not want to have an extended gentle CS (*n* = 73) most often cited their fear that they or their partner would feel overwhelmed by the experience (*n* = 30) or that they had no need for it (*n* = 13). Women said, they would not “want to see my own belly cut open”, and they were afraid or felt unsure if they could deal with seeing much blood. Some women mentioned that they had not been informed about this option and that if they had been informed, they would have opted for it (*n* = 4).

## Discussion

In a bid to improve women’s experiences of having a CS, we introduced an extended gentle CS protocol by encompassing the possibility of visual contact with the baby as it is being delivered via a drape that features a transparent part or window. Inferential statistics did not show any advantage or disadvantage attached to the visual contact in terms of birth satisfaction and fulfilled expectations. Quality of care, the type of CS (planned or unplanned) and early skin-to-skin contact had a greater overall impact. In addition, the development of the mother-to-child bond and the duration of breastfeeding seemed to depend on many additional factors. However, descriptive statistics (cf. Table 3) revealed that women who experienced an extended CS clearly preferred this method for any future CS. None of these women reported that they felt overwhelmed by the exposure. Of all other women, about half would be willing to try a CS with a transparent drape allowing for visual contact.

In summary, the extended gentle CS appears to be a viable option for many women, but our study did not result in a measurable benefit in terms of various outcomes. Birth satisfaction and other psychosocial outcomes most likely depend on a complex interplay between several aspects of the CS, early skin-to-skin contact and the possibility of observing the delivery, but also many other individual factors, such as childbirth expectations and fear of childbirth. A much larger and more homogeneous sample would certainly have led to more precise effect estimates. Accordingly, we cannot conclusively assess the impact of extended gentle CS on these outcomes. Further limitations of this study include a low response rate. Although there was no indication that this led to a bias between the two groups (gentle CS vs. extended gentle CS), the sample size is small such that the results should be considered as preliminary. For many women the birth had also taken place more than one year ago. Whilst a study using Salmon’s Item List has shown that measures of overall satisfaction with childbirth can be quite stable, at least until the second year after childbirth [19], it has also been argued that assessments of childbirth may come “too soon” [20].

Early skin-to-skin contact in the operating room after a CS with healthy and mature newborns is a widespread procedure in Switzerland. Our results suggest that—when compared with visual contact with the baby—early skin-to-skin contact in the operating room has a larger impact on the overall birth experience. In addition, other studies suggest that early physical contact has a positive influence on the mother’s mental well-being and on the mother-to-child bond [21, 22]. In the current study, mothers with premature births rated their bond with their child less positively. As extended paediatric care is needed for sick or premature newborns, they must be separated from their parents immediately, therefore bonding is not possible. Daily practice experience suggests that the visual contact as part of the extended gentle CS may be particularly beneficial in high-risk obstetric care, in situations when early skin-to-skin contact may not be possible.

Our data showed that the quality of care provided by healthcare professionals was most strongly associated with overall birth satisfaction. This result confirms findings from other studies that have shown that information being provided about the procedure and active involvement in the birth process significantly improve the birth experience [14, 23], and it suggests that involvement and maternal control is not only relevant in vaginal births with uncertain courses, but also in CS [4, 23–26]. Moreover, some women in our study also mentioned that they felt more actively involved by having an insight into the birth process.

In terms of implementing a gentle or an extended gentle CS protocol, strict delineation of responsibilities, roles and rituals in the operating room is an impediment [27, 28]. Lack of antenatal education of staff and parents, understaffing, time pressure and lack of equipment were also identified as obstacles to intraoperative skin-to-skin contact [29, 30]. In line with these reports, our results show that implementation can still be improved, as many women felt that they would have liked to have seen more of the baby or have had longer visual contact. The thin line between a too early, at worst traumatising sight, and the women's desire to see as much as possible, shows the importance of well-coordinated interdisciplinary collaboration in preparing the women for CS and good communication between the teams in the operating room. Future studies should therefore also investigate whether live transmission with cameras and a screen could simplify these issues around the timing and the angle of the view.

Extended gentle CS were more likely to be performed as planned CS and more often on women with a higher level of education, a higher insurance class and fewer preterm births. Accordingly, this subsample consists of women who are well educated, who may better understand the implications of their choice, and are therefore less intimidated by it. In daily practice, the fear of seeing the open abdomen was the most commonly cited reason for declining the procedure. In the current study, about 40% of those who had a gentle CS thought they would be overwhelmed by the experience.

In conclusion, the present work should be seen in the context of efforts to improve the birth experience for these women in particular, to strengthen their feeling of involvement and to facilitate the mother-to-child bond. Although this study reports only preliminary results, showing no advantage for the extended gentle CS, we suggest that both intraoperative skin-to-skin contact and a transparent drape should be offered to all women for whom there are no contraindications.

## Supplementary Information

Additional File 1 (PDF 251 kb)

## Data Availability

The final dataset used for quantitative analyses is available from the corresponding author on reasonable request.
