# Supplementary material for: The extended gentle caesarean section protocol—expanding the scope and adding value for the family: a cross-sectional study
Source: Arch Gynecol Obstet. 2023 Jan 16;307(5):1481–8. doi: 10.1007/s00404-023-06913-0 (PMC10110671; doi:10.1007/s00404-023-06913-0)
Supplement: Supplementary file 1 — Additional File 1 (PDF 251 kb) [file 404_2023_6913_MOESM1_ESM.pdf]

Additional File 1: The final questionnaire, originally developed in German and translated for the purpose of this publication.

Comment: Information provided in [square brackets] were not shown to participants.

1. [general section]

We will now ask you various questions about the birth of your child. If you have given birth more than once, these questions will always **relate to your experience of the last** birth by cesarean section.

1.1 **When** was it decided that you should have a cesarean section?

- ○ The cesarean section was already planned before going to the hospital.
- ○ After delivery had already begun.
- ○ I required an emergency cesarean within 15 minutes.

1.2 [Salmon's Item List, German short (Stadlmayr et al., 2009)]

1.3 How do the following statements apply to the **cesarean section you experienced**?

	strongly disagree	disagree	neutral	agree	totally agree
I felt the staff was working well together, before, during and after the cesarean section.	○	○	○	○	○
The cesarean section matched my expectations.	○	○	○	○	○
At the time, I understood the decision to have a cesarean section.	○	○	○	○	○

1.4 How much do you agree with the following statements **concerning care provided by professionals**?

	strongly disagree	disagree	neutral	agree	totally agree
The professionals informed me sufficiently **during** birth.	○	○	○	○	○
I was informed about all important procedures **before** the cesarean section.	○	○	○	○	○
I felt that the healthcare professionals were open to my wishes and needs.	○	○	○	○	○
**During** birth, I felt actively involved.	○	○	○	○	○

1.5 Was this a **multiple birth**?

◯ Yes, I had twins, triplets, etc.
◯ No

1.6 [Information conditional on 1.5 = "Yes, I had twins, triplets, etc."]

You stated that you had a multiple birth. From this point on, please assume all questions about "your child" refer to all of the children you gave birth to at the end of your most recent pregnancy.

1.7 Were you able to hold your baby with direct skin-to-skin contact already in the operating room?

◯ Yes
◯ No

1.8 How much do you agree with the following statement?

	strongly disagree	disagree	neutral	agree	totally agree
In the first hours after the cesarean section, my child was still a stranger to me.	◯	◯	◯	◯	◯
After the cesarean section, it was easy to form a bond with my child.	◯	◯	◯	◯	◯

1.9 Did you breastfeed?

◯ Yes, I still do.
◯ Yes, I did.
◯ No

1.10 How long did you breastfeed, completely or partially?
[question conditional on 1.9 = "Yes, I did."]

◯ less than 1 month
◯ 1 month
◯ …
◯ 12 months
◯ more than 12 months

1.11 Did you want to breastfeed?
[question conditional on 1.9 = "No"]

◯ Yes, but it wasn't possible for the following reasons: _________________
◯ No, I did not want to breastfeed.

1.12 How many hours after the cesarean section did you hold your baby to the breast for the first time?
[question conditional on 1.9 ≠ “No”]

- ◯ less than 1 hour
- ◯ 1 hour
- ◯ …
- ◯ 12 hours
- ◯ more than 12 hours

2. Gentle cesarean section

2.1 [Definition gentle cesarean section]

Since 2017, the University Women's Hospital of Bern offers the option of a **gentle cesarean section.** The procedure includes a transparent drape between patient and surgeon. The mother and her partner can observe the cesarean section if they wish to.

2.2 Did you have such a gentle cesarean section?

- ◯ Yes
- ◯ No

2.2.1 In hindsight, how do **you** rate the view through the transparent drape?
[question conditional on 2.2 = “Yes”]

- ◯ I would have preferred not to see that much of the birth.
- ◯ It was just right how much I saw from the birth.
- ◯ I would have preferred to see more of the birth.

2.2.2 If **your partner** was with you during the cesarean section, what do you think, how he/she would rate the view through the drape?
[question conditional on 2.2 = “Yes”]

- ◯ He/she would have preferred to see less of the birth.
- ◯ It was just right how much he/she saw from the birth.
- ◯ He/she would have preferred to see more of the birth.
- ◯ I had no birth companion during the caesarean section.

2.2.3 Please describe how you experienced the gentle cesarean section as a whole process.
[question conditional on 2.2 = “Yes”]

____
____

2.2.4 In your opinion, how could we improve the birth experience during the gentle cesarean section?
[question conditional on 2.2 = “Yes”]

____
____

2.2.5 Why did you choose a gentle cesarean section?
[question conditional on 2.2 = “Yes”]

____
____

2.3 If another cesarean section would be performed at a next birth, which type would you prefer?

- ◯ Gentle cesarean section
- ◯ Standard cesarean section

2.4 Why?

____
____

3. [birth history / demographics]

3.1 Was this your first birth?

- ◯ Yes
- ◯ No

3.2 **For births prior to this most recent cesarean section only**, please indicate if it was a cesarean or vaginal birth.
[question conditional on 3.1 = "No"]

	Mode of birth	
	Cesarean section	Vaginal birth
1st birth	◯	◯
2nd birth	◯	◯
3rd birth	◯	◯

3.3 Did you breastfeed your children fully or partially? Again, indicate the duration of breastfeeding **only for the children** you gave birth to **before the last cesarean section.**
[question conditional on 3.1 = "No"]

	Duration of breastfeeding					
	did not breastfeed	less than 1 month	1 month	…	12 months	more than 12 months
Child/ren from 1st birth	◯	◯	◯	◯	◯	◯
Child/ren from 2nd birth	◯	◯	◯	◯	◯	◯
Child/ren from 3rd birth	◯	◯	◯	◯	◯	◯

3.4 [Whooley questions (Whooley et al., 1997)]

3.5 What is your highest degree of education?

- ◯ Compulsory education
- ◯ Apprenticeship
- ◯ Grammar school, vocational A level, specialised secondary school certificate (FMS), vocational college (DMS)
- ◯ Higher technical and vocational training
- ◯ University of Applied Sciences, educational college
- ◯ University, Federal Institute of Technology (EPFL, ETH)
- ◯ Other: ____

3.6 You completed the questionnaire. Is there anything you would like to let us know?

____

References

Stadlmayr W, Bitzer J, Hösli I, Amsler F, Leupold J, Schwendke-Kliem A, et al. Birth as a multidimensional experience: comparison of the English- and German-language versions of Salmon's item list. J Psychosom Obstet Gynecol. 2009;22(4):205–14. https://doi.org/10.3109/01674820109049975.

Whooley MA, Avins AL, Miranda J, Browner WS. Case-finding instruments for depression. Two questions are as good as many. J Gen Intern Med. 1997;12(7):439–45. https://doi.org/10.1046/j.1525-1497.1997.00076.x.
